# Additional radiotherapy following endoscopic submucosal dissection for T1a-MM/T1b-SM esophageal squamous cell carcinoma improves locoregional control

**DOI:** 10.1186/s13014-018-0960-y

**Published:** 2018-01-29

**Authors:** Osamu Hisano, Takeshi Nonoshita, Hidenari Hirata, Tomonari Sasaki, Hideyuki Watanabe, Hiroaki Wakiyama, Minoru Ono, Saiji Ohga, Hiroshi Honda

**Affiliations:** 1 Department of Clinical Radiology, Kyushu University Graduate School of Medical Sciences, 3-1-1 Maidashi, Higashi-ku, Fukuoka, 812-8582 Japan; 2 Department of Radiology, Kitakyushu Municipal Medical Center, 2-1-1 Bashaku, Kokurakita-ku, Kitakyushu, 802-0077 Japan

**Keywords:** Esophageal squamous cell carcinoma, Endoscopic submucosal dissection, Additional radiotherapy, Esophageal stricture

## Abstract

**Background:**

Endoscopic submucosal dissection (ESD) can be used as a less invasive treatment option for superficial esophageal cancer involving the muscularis mucosae (T1a-MM) or upper third of the submucosa (T1b-SM1). Additional treatment after ESD is needed to prevent lymph node metastasis. However, the efficacy of radiotherapy following ESD has not been well evaluated. Moreover, the clinical outcomes of patients with large mucosal defects of the esophagus who received radiotherapy after ESD have not been reported. This study aimed to clarify the efficacy of additional radiotherapy following ESD for esophageal squamous cell cancer involving T1a-MM or T1b-SM1.

**Methods:**

We analyzed twenty-seven patients with pathologically confirmed T1a-MM or T1b-SM1 esophageal squamous cell cancer treated by ESD. Thirteen patients received additional radiotherapy (RT group), and the remaining patients did not (non-RT group). Locoregional control (LRC), overall survival, cause-specific survival, and adverse events including treatment-related esophageal strictures were evaluated.

**Results:**

The three-year LRC was significantly better for the RT than the non-RT group (100% vs. 57.8%, respectively; *p* = 0.022). Chemotherapy following ESD did not improve LRC. Multivariate analysis showed that radiotherapy was an independent prognostic factor for better LRC (*p* = 0.0022). Contrary to the results in LRC, overall and cause-specific survival were not significantly different between the RT and non-RT groups. A subgroup analysis of patients with mucosal defects involving ≥ 3/4 of the esophageal circumference after ESD showed that LRC of the RT group was better than that of the non-RT group (*p* = 0.049). Treatment-related esophageal strictures were observed in 2 of 6 patients in the RT group with large mucosal defects after ESD. No patients with mucosal defects involving less than 3/4 of the circumference after ESD developed treatment-related strictures.

**Conclusions:**

Radiotherapy after ESD contributed to better LRC in esophageal squamous cell cancer involving pT1a-MM and pT1b-SM1. Esophageal strictures were observed in some patients with large mucosal defects after ESD. Despite leading to better LRC, radiotherapy after ESD should be undertaken after careful consideration for patients with large mucosal defects after ESD.

## Background

The choice of therapy for superficial esophageal squamous cell cancer (ESCC) depends on the risk of lymph node metastasis [1]. Pathological T1a-intraepithelial cancer or pT1a-cancer within the lamina propria mucosae has a very low risk of lymph node metastasis; therefore these cancers are potentially curable by endoscopic resection alone [2, 3]. On the other hand, the incidence of lymph node metastases in ESCC involving the muscularis mucosae (pT1a-MM) and the upper third of the submucosa (less than 200 μm below the MM; pT1b-SM1) is reported to range from approximately 4.2% to 30% [2, 4]. The standard treatment for ESCC involving pT1a-MM or pT1b-SM1 is surgical resection. However, because of the relatively low risk of lymph node metastasis, endoscopic resection can be used as a less invasive treatment option [4]. Endoscopic submucosal dissection (ESD), an advanced form of endoscopic resection, enables *en bloc* resection of a larger mucosal lesion and more precise histological assessment compared with endoscopic mucosal resection (EMR) [3]. ESD for ESCC involving T1a-MM or T1b-SM1 is performed as a diagnostic resection or for inoperable patients. This technique is usually performed for lesions occupying less than three-quarters of the circumference of the esophagus, because the incidence of esophageal stricture after ESD is low [5]. Considering the incidence of lymph node metastases in ESCC involving pT1a-MM or pT1b-SM1, additional treatment is needed after ESD to prevent lymph node metastasis [4]. Only a few studies have reported the efficacy of additional radiotherapy (RT) performed after endoscopic resection for ESCC involving pT1a-MM or pT1b-SM1 [6–8]. However, to our knowledge, no study has compared the efficacy of ESD with additional RT to the efficacy of ESD without RT. Moreover, the clinical outcomes of patients with mucosal defects involving three-quarters or more (≥ 3/4) of the esophageal circumference who received RT after ESD have not been reported. We previously reported that RT also induces treatment-related esophageal stricture [9, 10]. It is possible that RT affect the development of treatment-related esophageal stricture in the patients with large mucosal defects after ESD.

The aim of our study was to clarify the efficacy of RT after ESD for pT1a-MM or pT1b-SM1 ESCC by investigating locoregional control and survival in patients with ESCC treated by ESD with or without additional RT. Furthermore, since the mucosal defects after ESD were ≥ 3/4 of the circumference of the esophagus in some patients, we estimated the effect of RT added to ESD on the development of treatment-related esophageal stricture.

## Methods

### Patients

This retrospective analysis was approved by the Institutional Review Board of the Kitakyushu Municipal Medical Center. From March 2005 to December 2014, consecutive 32 patients with pathologically confirmed T1a-MM or T1b-SM1 ESCC treated by ESD were enrolled. Three and 2 patients with ESCC occurring in double primary and triple primary sites, respectively, underwent *en bloc* resection by ESD. All the patients had stage IA disease (pT1aN0M0 or pT1bN0M0), according to the Seventh Edition of the International Union against Cancer TNM classification system. Patients who received surgical treatment after ESD (*n* = 2), who had previously received definitive RT for ESCC (n = 2), and who were lost to follow up (*n* = 1), were excluded from this study. Therefore, a total of 27 patients were eligible. All the tumors were in the thoracic esophagus. The median duration of follow-up for 27 patients was 40.3 months (range, 6.0–152.2 months).

### Radiotherapy and chemotherapy

ESD with or without additional RT was used for their initial treatment for ESCC. None of the eligible patients had received RT, chemotherapy or surgery for ESCC. Among the 27 patients treated by ESD, 13 patients received RT in addition to ESD. RT was administered within 2 months after ESD, with the exception of 1 patient who received RT 6 months after ESD because of an esophageal stricture developing after ESD. The patients underwent three-dimensional conformal radiotherapy delivered by a Siemens Oncor linear accelerator (Siemens Healthcare, Erlangen, Germany) with a 6–10 MV X-ray beam. The initial treatment dose consisted of 40–41.4 Gy (median 40 Gy, 1.8–2 Gy per fraction), which was delivered to the following regional lymph nodes. The clinical target volume 1 (CTV1) of the 13 patients receiving RT was defined as follows: whole mediastinum for middle thoracic ESCC (*n* = 5); region of supraclavicular to middle thoracic paraesophageal lymph nodes for upper thoracic ESCC (*n* = 3); supraclavicular to pericardial region for middle thoracic ESCC (*n* = 2); and upper thoracic to pericardial region for lower thoracic ESCC (n = 2). The radiation boost, CTV2, was generated based on approximately 20-mm longitudinal margins of the estimated tumor bed. The planning target volumes (PTVs) were calculated by adding 5-mm margins to each CTV (PTV1, PTV2). Following a dose of 40–41.4 Gy to the PTV1, a boost dose was administered to the PTV2. A total dose of 61.4 Gy was administered to 1 patient who had positive resection margins. Seven patients with negative resection margins received a final total dose of 50–50.4 Gy (median 50 Gy). The remaining 5 patients did not receive boost irradiation, and therefore received a final total RT dose of 40–41.4Gy. RT was completed without interruption for all 13 patients. Dose constraints for the major organs at risks were as follows: maximal dose to the spinal cord, < 50 Gy; mean dose to the heart, < 40 Gy; and percentage of total lung volume receiving ≥20 Gy, < 20%.

Additional chemotherapy was administered to 8 patients after ESD, 4 of whom received concurrent radiotherapy. The drug regimens of the patients receiving concurrent chemoradiotherapy were as follows: 5-fluorouracil plus cisplatin (*n* = 2), and tegafur/gimeracil/oteracil potassium (S-1) (*n* = 2). Four patients not administered RT received additional chemotherapy as follows: 5-fluorouracil plus cisplatin (*n* = 1), carboplatin plus docetaxel (*n* = 1), S-1 (*n* = 1), and S-1 plus cisplatin (*n* = 1).

### Follow up and evaluation

Patients were followed every 3–6 month for 3 years. They were tested for serum tumor markers, and underwent computed tomography (CT) and gastrointestinal endoscopy. Locoregional recurrence was defined as recurrence of the primary tumor or metastases to the locoregional lymph node observed on endoscopy or CT. The duration of locoregional control (LRC) was calculated from the date of ESD to the first observation of locoregional recurrence. Cause-specific survival (CSS) was defined as the time to death of ESCC. CSS and overall survival (OS) were also calculated from the date of ESD. All adverse events, including treatment-related esophageal stricture, were recorded according to the Common Terminology Criteria for Adverse Events, version 4.0.

### Statistical analysis

Continuous variables were analyzed by the Mann-Whitney U test. Categorical variables were analyzed by the Fisher exact test. LRC, OS, and CSS were estimated using the Kaplan-Meier method. Survival curves were compared using the log-rank test. A two-sided *p*-value less than 0.05 was considered significant. Multivariate analysis for LRC was performed using Cox proportional hazards models. Variables with a *p*-value less than 0.2 in univariate analysis were included in the multivariate analysis. Data analysis was performed using JMP 11 (SAS Institute Inc., Cary, NC, USA) and R version 3.2.4 (The R Foundation) software.

## Results

### Patient characteristics

Twenty-seven patients with pT1a-MM or pT1b-SM1 ESCC were divided into 2 groups. Thirteen patients who received additional RT after ESD were classified as the RT group. The median time elapsed from the end of ESD to the starting date of RT was 2.3 months (range, 0.8–6.3 months). The remaining 14 patients without additional RT were classified as the non-RT group. The reasons for not performing RT after ESD were as follows: patient refusal (*n* = 5), old age (*n* = 1), esophageal stricture after ESD (*n* = 1), presence of synchronous laryngeal cancer (*n* = 1), history of RT for breast cancer (*n* = 1), and physician decision (*n* = 5). Patient characteristics are listed in Table 1. Differences in the clinicopathological factors of patients, including positive resection margins, between the RT and non-RT groups were not significant. Mucosal defects involving ≥ 3/4 of the esophageal circumference after ESD were observed in 6 patients in the RT group and 4 in the non-RT group, which was not significantly different (*p =* 0.44). Three patients underwent complete circumferential dissection, 2 of whom were administered RT at doses of 50.4 Gy to 61.4 Gy. Differences in the mean longitudinal diameters of the mucosal defects (*p =* 0.88) and the number of patients receiving chemotherapy (*p =* 1.00) were not significant between groups.

**Table 1:** Clinicopathological factors of patients treated by ESD with or without additional RT (*n* = 27)

Factors		RT group (*n* = 13)	Non-RT group (*n* = 14)	*p-*value
Age, yr. (mean ± SD)		66 ± 5	68 ± 7.4	0.31
Gender	Male	10	12	0.65
	Female	3	2	
Number of primary tumors	Single	12	10	0.36
	Multiple	1	4	
Location of tumor	Ut	3	2	0.59
	Mt	8	11	
	Lt	2	1	
Invasion depth	pT1a-MM	6	10	0.25
	pT1b-SM1	7	4	
Circumferential extent of ESD	< 3/4	7	10	0.44
	≥3/4	6	4	
Longitudinal diameter of ESD (mm; mean ± SD)		43 ± 17	42 ± 21	0.88
Positive resection margin		1	0	1.00
Positive lymphatic invasion		1	0	0.48
Positive vascular invasion		0	0	1.00
Additional chemotherapy	(+)	4	4	1.00
	(−)	9	10	

*ESD* endoscopic submucosal dissection, *RT* radiotherapy, *Ut* upper thoracic tumor, *Mt.* middle thoracic tumor, *Lt* lower thoracic tumor, *T1a-MM* tumor invading muscularis mucosae, *T1b-SM1* tumor invading the upper third of the submucosa

### Additional radiotherapy improved locoregional control but not survival

**Fig. 1:**
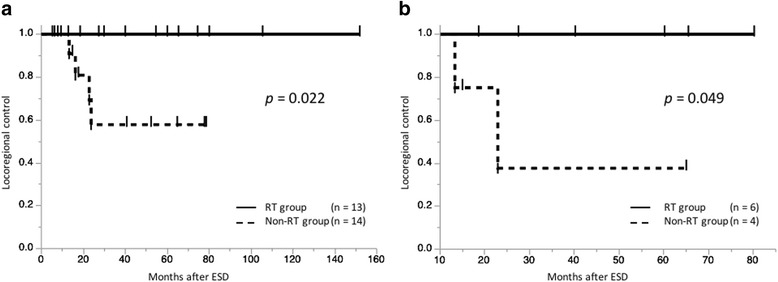
Kaplan-Meier survival curves for locoregional control (LRC). LRC after endoscopic submucosal dissection (ESD) with or without additional radiotherapy (RT) for all study patients with esophageal squamous cell cancer stratified by whether or not they received RT (**a**) and survival curves of patients with ESD-related mucosal defects ≥ 3/4 of the esophageal circumference with or without additional RT (**b**)

None of the patients in the RT and non-RT groups showed recurrence of the primary tumor. No regional lymph node recurrence was detected in the RT group. On the other hand, 4 of 14 patients (29%) in the non-RT group developed regional lymph node recurrences, 2 of whom received additional chemotherapy (5-fluorouracil plus cisplatin). Notably, 2 patients with pT1a-MM ESCC and negative resection margins and no lymphovascular invasion, who were considered good candidates for ESD without any additional therapy [11], developed regional lymph node recurrence. Salvage treatments for recurrence in 4 patients in the non-RT group were as follows: 1 patient with paraesophageal nodes recurrence underwent surgery; 2 patients with right supraclavicular or gastric nodes recurrence underwent chemoradiotherapy (60–66 Gy); and 1 patient with paraesophageal node recurrence underwent chemoradiotherapy (66 Gy) followed by surgery. The median time from the date of ESD to regional lymph node recurrence was 19.5 months (range, 13–24 months). The 3-year LRC rate of the RT group was significantly better than that of the non-RT group (100% vs. 57.8%, *p =* 0.022; Fig. 1a). Multivariate analysis showed that additional RT was an independent prognostic factor for better LRC (*p =* 0.0022; Table 2). Additional chemotherapy following ESD did not improve LRC by univariate analysis, and rather tended to poorer LRC by multivariate analysis. A subgroup analysis of patients with mucosal defects involving ≥ 3/4 of the esophageal circumference after ESD showed that LRC for the RT group was better than that for the non-RT group (*p =* 0.049; Fig. 1b).

**Table 2:** Univariate and multivariate analysis for locoregional control

				Univariate analysis	Multivariate analysis
Factors		Number	3-year LRC (%)	*p-*value	*p-*value
Age, yr	≥65	18	86	0.51	(−)
	< 65	9	67		
Gender	Male	22	78	0.46	(−)
	Female	5	100		
Number of primary tumors	Single	22	82	0.48	(−)
	Multiple	5	75		
Invasion depth	pT1a-MM	16	71	0.24	(−)
	pT1b-SM1	11	90		
Circumferential extent of ESD	< 3/4	17	83	0.71	(−)
	≥3/4	10	77		
Longitudinal diameter of ESD (mm)	< 40	13	76	0.77	(−)
	≥40	14	83		
Additional chemotherapy	(+)	8	60	0.16	0.039*
	(−)	19	87		
Additional RT	(+)	13	100	0.022*	0.0022*
	(−)	14	58		

*ESD* Endoscopic submucosal dissection, *RT* Radiotherapy, *LRC* Locoregional control, *pT1a-MM* Tumor invading muscularis mucosae, *pT1b-SM1* Tumor invading submucosa less than 200 μm below the MM. *p < 0.05

With regard to survival, 2 of 13 patients in the RT group died of ESCC. One of the patients with pT1b-SM1 ESCC, who was treated by chemoradiotherapy (50.4 Gy with 5-fluorouracil plus cisplatin), died of cytologically proven meningeal dissemination 3 years after treatment. The other patient with pT1a-MM ESCC and lymphatic invasion, who received RT at a total dose of 41.4 Gy, died of multiple liver metastases that developed 5 months following treatment. In the non-RT group, 2 of 14 patients died of ESCC recurring in the regional lymph nodes. The 3-year CSS and OS of the RT and non-RT groups were as follows: CSS, 82.1% vs. 77.8% (*p =* 0.80), respectively; and OS, 67.1% vs. 65.8% (*p =* 0.87), respectively. Of the 13 patients with double cancers, cancers affecting the clinical outcome were seen for 2 patients with metachronous hypopharyngeal cancers (1 in the RT group and 1 in the non-RT group). Although they were treated with chemoradiotherapy for hypopharyngeal cancers after they received treatment for ESCC, both died of hypopharyngeal cancer. Overall, our findings indicate that additional RT was significantly associated with better LRC but not with survival in patients with pT1a-MM or pT1b-SM1 ESCC treated with ESD.

### Treatment-related esophageal stricture and other adverse events

Esophageal stricture is a common adverse event of endoscopic resection, and it occurs frequently in patients with mucosal defects involving ≥ 3/4 of the esophageal circumference after endoscopic resection [4, 5]. Of the patients with mucosal defects involving ≥ 3/4 of the circumference after ESD, 2 of 6 patients in the RT group (33%) and 2 of 4 in the non-RT group (50%) developed esophageal strictures. Regardless of whether patients received RT after ESD or not, no patients with mucosal defects involving < 3/4 of the circumference after ESD developed treatment-related strictures. The characteristics of patients with treatment-related esophageal stricture are shown in Table 3. Among the 27 patients with pT1a-MM or pT1b-SM1 ESCC, the difference in the incidence of esophageal stricture between the RT and non-RT groups was not significant (*p =* 1.00; Table 4). In addition, the patients who developed esophageal strictures after ESD had mucosal defects with longer longitudinal diameters (*p* = 0.0033), plus higher rate of multiple primary tumors (*p* = 0.013). Grade 3 strictures were observed in 2 patients (1 in the RT group and 1 in the non-RT group); both underwent complete circumferential resection for ESCC. One patient in the non-RT group received continuous endoscopic balloon dilatation (EBD) until dying of ESCC. Another patient in the RT group underwent esophageal stent placement following EBD. The grade 2 esophageal strictures in 2 patients were manageable by medication and EBD.

**Table 3:** Patients with treatment-related esophageal strictures (*n* = 4)

Additional RT	Toxicity grade	Invasion depth	Circumference of ESD^a^	Longitudinal diameter of ESD (mm)	Treatment of strictures	Outcome of strictures
(+)	Grade 2	pT1a-MM	5/6	68	EBD	Improved
(+)	Grade 3	pT1b-SM1	Complete	75	EBD, stent	Remained
(−)	Grade 2	pT1a-MM	3/4	55	EBD	Improved
(−)	Grade 3	pT1a-MM	Complete	90	EBD	Remained

^a^No patients with mucosal defects involving less than 3/4 of the circumference after ESD developed treatment-related strictures

*RT* radiotherapy, *ESD* endoscopic submucosal dissection, *T1a-MM* tumor invading muscularis mucosae, *T1b-SM1* tumor invading the upper third of the submucosa, *EBD* endoscopic balloon dilatation

**Table 4:** Factors associated with treatment-related esophageal stricture

Factors		Esophageal stricture (*n* = 4)	No esophageal stricture (*n* = 23)	*p-*Value
Age (years; mean ± SD)		65 ± 6.5	67 ± 1.4	0.61
Gender	Male	2	20	0.14
	Female	2	3	
Number of primary tumors	Single	1	21	0.013*
	Multiple	3	2	
Invasion depth	pT1a-MM	3	13	0.62
	pT1b-SM1	1	10	
Circumferential extent of ESD	< 3/4	0	17	0.012*
	≥3/4	4	6	
Longitudinal diameter of ESD (mm; mean ± SD)		72 ± 14	38 ± 14	0.0033*
Additional chemotherapy	(+)	1	7	1.00
	(−)	3	16	
Additional RT	(+)	2	11	1.00
	(−)	2	12	

*ESD* Endoscopic submucosal dissection, *RT* Radiotherapy, *pT1a-MM* Tumor invading muscularis mucosae, *pT1b-SM1* Tumor invading submucosa less than 200 μm below the MM. *p < 0.05

Other serious adverse events were as follows: grade 3 radiation pneumonitis (*n* = 1) and grade 3 neutropenia during chemoradiotherapy (*n* = 2) in the RT group. Grade 2 esophageal perforation was observed immediately after ESD in 2 patients in the RT group and in 1 patient in the non-RT group. All these 3 patients improved with 2 to 5 days of fasting therapy and peripheral parental nutrition. One patient developed grade 2 pericardial effusion detected 2 years after RT. Collectively, these results suggest that, in patients with a mucosal defect after ESD of < 3/4 of the circumference, additional RT did not result in an increased incidence of treatment-related esophageal stricture. However, some patients with mucosal defects involving ≥ 3/4 of the circumference after ESD, in both RT group and non-RT group, had esophageal stricture. Other non-negligible side effects such as radiation pneumonitis were observed in a few patients who received RT.

## Discussion

The results of this study demonstrated the efficacy and safety of additional RT after ESD for patients with ESCC involving pT1a-MM or pT1b-SM1. RT following ESD contributed to better LRC than ESD without additional RT. On the other hand, the OS and CSS of the RT and non-RT groups did not differ significantly. Furthermore, despite good LRC, esophageal strictures were observed in some patients in the RT group with large mucosal defects after ESD.

A few studies have focused on the efficacy of RT or chemoradiotherapy after endoscopic resection (ESD or EMR) for ESCC involving pT1a-MM or pT1b-SM1. Similar to our findings, Shimizu et al. showed that none of 16 patients who underwent RT combined with 5-fluorouracil plus cisplatin after EMR developed local recurrence and metastases [8]. Moreover, ESD combined with chemoradiotherapy was reported to be associated with better LRC compared with chemoradiotherapy alone; however, only 6 cases were staged as pT1a-MM or pT1b-SM1 disease [6]. A recent prospective single-arm confirmatory study (Japan Clinical Oncology Group [JCOG] 0508) suggested that prophylactic chemoradiotherapy after endoscopic resection was an effective treatment for pT1a-MM ESCC with lymphovascular invasion or pT1b-SM1 ESCC without lymphovascular invasion [11, 12]. In other words, patients at low risk of recurrence such as pT1a-MM ESCC with negative resection margins and no lymphovascular invasion are considered to be good candidates for ESD without any additional therapy [4, 11]. Nevertheless, despite the small sample size of our study, it is notable that 2 such patients in the non-RT group developed regional lymph node metastases, which suggest that some of these cases will need additional RT after ESD.

In our study, additional RT was not found to contribute to longer OS or CSS compared with the non-RT patients. A few reasons might account for this finding. First, the survival rates of our patients were lower than the rates in other previous studies [6–8, 12], possibly because our study included cases that have often been excluded from clinical trials. These include inoperable patients, patients with double cancers, and cases undergoing almost or completely circumferential ESD. Second, some of the patients in the RT group did not receive any chemotherapy. And last, our study had limitations, including a small number of patients and relatively short follow-up period.

The efficacy and safety of RT following ESD that resulted in mucosal defects involving ≥ 3/4 of the esophageal circumference has not been previously reported, because such cases are often excluded from studies because of the risk of esophageal stricture. In our retrospective analysis, 6 patients underwent RT for medical reasons after ESD that had resulted in mucosal defects that involved ≥ 3/4 of the esophageal circumference. They achieved better LRC than patients without additional RT; however, severe esophageal strictures were observed in 2 of 6 patients. In addition to the circumference of the ESD, long longitudinal diameter of resection and the number of the primary tumors were related to the occurrence of esophageal stricture, consistent with findings in previous studies [4, 5]. The administration of RT to patients with mucosal defects involving ≥ 3/4 of the esophageal circumference should be undertaken with careful consideration, although the treatment-related esophageal strictures of our patients were all manageable.

## Conclusions

Our study found that additional RT after ESD contributed to better LRC in patients with ESCC involving pT1a-MM and pT1b-SM1 disease. In patients with mucosal defects after ESD that involve ≥ 3/4 of the esophageal circumference, some patients developed treatment-related esophageal strictures. Therefore, despite leading to better LRC, RT after ESD should be undertaken after careful consideration for patients with large mucosal defects after ESD.

## Abbreviations

CSS: Cause-specific survival
CT: Computed tomography
CTV: Clinical target volume
EBD: Endoscopic balloon dilatation
EMR: Endoscopic mucosal dissection
ESCC: Esophageal squamous cell cancer
ESD: Endoscopic submucosal dissection
LRC: Locoregional control
OS: Overall survival
PTV: Planning target volume
RT: Radiotherapy
T1a-MM: ESCC involving the muscularis mucosae
T1b-SM1: ESCC involving the upper third of the submucosa
